# Retraction Note: Repression of LncRNA PART1 attenuates ovarian cancer cell viability, migration and invasion through the miR-503-5p/FOXK1 axis

**DOI:** 10.1186/s12885-026-15605-2

**Published:** 2026-01-22

**Authors:** Bing Li, Ge Lou, Jiahui Zhang, Ning Cao, Xi Yu

**Affiliations:** 1 Department of Gynaecology, Harbin Medical University Cancer Hospital, No. 150, Haping Road, Nangang District, Harbin City, Heilongjiang Province 150081 China; 2 School of Basic Medical Sciences, Fudan University, No.138, Medical College Road, Shanghai, 200032 China

**Retraction Note: BMC Cancer 22, 124 (2022)**

**https://doi.org/10.1186/s12885-021-09005-x**

The Editors have retracted this article. After publication it was noted that the TUNEL assays presented in Fig. 7G have been previously published [1] in an article with no authors in common. The Editors no longer have confidence in the reliability of the presented work.

All authors agree with this retraction.
